# Adult Respiratory Syncytial Virus Infection and Hypoxic Cardiac Arrest—Coexistent or Causal? A Hypothesis-Generating Case Report

**DOI:** 10.3390/medicina58081121

**Published:** 2022-08-18

**Authors:** Sebastian Schnaubelt, Felix Eibensteiner, Marieke Merrelaar, Daniel Tihanyi, Robert Strassl, Christian Clodi, Hans Domanovits, Heidrun Losert, Michael Holzer

**Affiliations:** 1 Department of Emergency Medicine, Medical University of Vienna, 1090 Vienna, Austria; 2 Department of Pulmonology, Clinic Penzing, Vienna Healthcare Group, 1140 Vienna, Austria; 3 Division of Clinical Virology, Department of Laboratory Medicine, Medical University of Vienna, 1090 Vienna, Austria

**Keywords:** respiratory syncytial virus, cardiac arrest, cardiopulmonary resuscitation

## Abstract

Respiratory syncytial virus (RSV) is a well-known pathogen in paediatric patients. However, it also causes substantial morbidity and mortality in adults, posing a major healthcare problem. We present a patient with chronic pulmonary conditions and an acute RSV infection, thus leading to cardiac arrest (CA). We speculate that RSV as the causative agent for CA should be considered in post-resuscitation care. From a wider public health perspective, immuno-naivety for RSV caused by the coronavirus disease 2019 pandemic may induce a severe rise in cases, morbidity, and mortality in the future.

## 1. Introduction

Since the respiratory syncytial virus (RSV) of the pneumoviridae family was discovered in the mid-20th century, it has long played an ostensible underpart in the yearly “flu season” [1]. While it is known to cause substantial morbidity and even mortality among paediatric patients [2], RSV was, in recent decades, increasingly identified to also affect adults: Up to 12% of acute respiratory illnesses [1], and as much as 25% of the annual excess mortality during the winter months—historically attributed to influenza—are now thought to be caused by an RSV infection [3]. With an average illness duration of 10 days, a length of hospital stay of 3 to 6 days, an overall mortality rate of up to 8%, an intensive care unit (ICU)-admittance rate of 10–30%, and a necessity for mechanical ventilation in 3–17% of cases [1,4], RSV infections emerge as a major health problem.

On a cellular level, RSV infects respiratory epithelial cells—inflicting damage to the superficial airway lining—and mediates an overshooting immune response in the respiratory system [1,5,6]. Several genetic polymorphisms have been discussed to influence pathogenicity but are yet to be fully understood [7].

RSV spreads through droplets, it can survive for several hours on nonporous surfaces. Aerosolisation is probably less important, but the relevant protective gear is nonetheless highly recommended [1,8,9]. As seen in other respiratory viral diseases, symptoms range from cough or nasal congestion up to pneumonia and acute respiratory distress syndrome (ARDS; up to 13%) [1,7,10,11], with an incubation time of 3–7 days [5]. Dyspnoea seems to be the chief complaint in the admitted cases (up to 93%) [1], and radiographic findings vary from normal chest X-rays (up to 42%) to faint opacities and lobar consolidation [7,12]. Asymptomatic courses are rare (<5%), and clinically, an RSV infection is hardly distinguishable from similar diseases. Bacterial or viral superinfection is common, leading to more severe courses and increased mortality [1]. Other complications include myocarditis or an exacerbation of pre-existing comorbidities such as congestive heart failure, asthma, or chronic obstructive pulmonary disease (COPD)—reports even suggest that, in the viral season, around 5% of hospital admissions for the worsening of congestive heart failure are in fact attributable to RSV [1,7,13,14]. Patients admitted due to RSV infections show underlying chronic pulmonary or cardiovascular conditions more often, compared with influenza cases [1]. Advanced age seems to be a particular risk factor for severe courses and mortality [7,14,15,16]; in fact, RSV was first described as a major health problem in adults after an outbreak in nursing homes in the 1970s [7].

The estimated annual healthcare cost of RSV disease is quite substantial, costing over half a billion US dollars for hospitalisations in the United States alone, and an unknown amount for additional outpatient treatment [17]. Increased airway reactivity after an infection can last up to several months, further impacting healthcare systems and economies due to potential follow-up visits and extended sick leaves [7,10].

Treatment generally consists of symptom-oriented and supportive measures, as well as management of complications including bacterial superinfection. The use of corticosteroids is still debated in the literature [7,18]. A variety of further therapeutic options including ribavirin or immunoglobulins have been suggested—with mixed results concerning efficacy and practicability. Novel antiviral agents such as fusion inhibitors can be promising future options [1,5,19]. However, a significant proportion of affected patients present late in the course of the disease, days after symptom onset, potentially rendering various therapies less efficacious [1]. Strategies to prevent symptomatic RSV infection include monoclonal antibodies, immunoglobulins, nanobodies, and—naturally—vaccines, with the latter still under development [1,20].

## 2. Surroundings and Materials

### 2.1. Setting and Patient

We reviewed a patient suffering from cardiac arrest (CA) and acute RSV who was admitted to the Department of Emergency Medicine, Medical University of Vienna, Austria, in November 2021. The respective emergency department (ED) is part of a tertiary academic hospital and consists of an outpatient department and an adjacent intermediate- and intensive care unit (IMCU/ICU). It is an accredited high-volume CA centre and treats approximately 300 CA cases per year. The patient’s clinical, imaging, and laboratory data were retrospectively assessed.

### 2.2. Viral Diagnosis

Testing for the presence of severe acute respiratory syndrome coronavirus 2 (SARS-CoV-2)-, influenza-, and RSV-RNA in nasopharyngeal respiratory specimens was performed via real-time qPCR, the recommended method of RSV detection. [1] Automated nucleic acid extraction from respiratory specimens was performed using the Roche MagNaPure 2.0 platform (Roche Diagnostics AG, Industriestrasse 7, 6343 Rotkreuz, Switzerland). TaqMan-based qualitative RT-PCR was performed using a LightCycler Multiplex RNA Virus Master (Roche Diagnostics, Rotkreuz, Switzerland) on a Roche LighCycler 480II cycler (Roche Diagnostics AG, Industriestrasse 7, 6343 Rotkreuz, Switzerland). Primers and probes were used as published elsewhere [21]. The following cycling protocol was applied: 8 min at 53 °C, 30 s at 95 °C, followed by 50 cycles of 1 s at 95 °C, 40 s at 60 °C, 1 s at 72 °C, and 30 s at 40 °C. Positive results (Ct value < 30) were confirmed by repeated testing.

## 3. Case Description

### 3.1. Pre-Hospital Scene

A 74-year-old male patient (body mass index 22.9) was found by a relative lying on the floor and gasping. Several hours before, he had complained about dyspnoea. Chest compressions were initiated by the relative but then abandoned to call for help. Emergency medical services (EMSs) including an emergency physician arrived 7 min later at the scene and provided advanced life support (ALS). Cardiopulmonary resuscitation (CPR) was continued by the EMS. No or low-flow timeframes could only be estimated (the time point of CA unclear) at around 7 to 15 min. Rhythm checks initially and subsequently showed pulseless electrical activity (sinus bradycardia). A cumulative dose of 1 mg of epinephrine was administered through peripheral venous access. The airway was secured via endotracheal intubation at the first attempt, without complications. After 6 min of CPR, a sustained return of spontaneous circulation (ROSC) was achieved (end-tidal carbon dioxide (etCO_2_) at ROSC 45 mmHg), and the patient was transported to the ED sedated and mechanically ventilated.

### 3.2. Arrival at the ED

The correctly intubated patient (pressure-controlled ventilation at 8 PEEP and 16 mmHg driving pressure, 100% FiO_2_) was admitted in a hypotonic state. A thorough physical exam revealed bilateral coarse respiratory sounds and a previously performed amputation of the right lower leg. The initial electrocardiogram (ECG) showed a sinus rhythm at 70 beats per minute with biphasic T-waves in V3-4; for the initial arterial blood gas analysis, see Table 1. Point-of-care ultrasound revealed a highly reduced left ventricular function and bilateral pleural effusions. Invasive arterial blood pressure monitoring as well as central venous access were established. Subsequently, norepinephrine support (0.05 µg/kg/min), an antiobstructive regimen including intravenous terbutalinsulfate, nebulised fenoterolhydrobromid/ipratropiumbromide, and 250 mg of prednisolone were given. Furthermore, continuous sedation via propofol and remifentanil, a fluid and electrolyte alternative, and prophylactic anticoagulation through 4000 international units of enoxaparin were administered. Additionally, we commenced targeted temperature management (TTM) following the local protocol (ArticSun^®^ (Medivance Corp, Louisville, CO, USA), target temperature 33 °C for 24 h). The catecholamine demand gradually declined, and circulatory support was no longer needed at 4 h after admission. An empirical antibiotic regimen with piperacillin/tazobactam (4 g 3 times daily) was established.

### 3.3. Workup of Past Medical History

A newly detected (October 2021) case of bronchial carcinoma (T1N0M0) with further diagnostics pending, and multiple other comorbidities that had partly not been sufficiently diagnosed were found: heart failure with reduced ejection fraction (no further details available), chronic obstructive pulmonary disease (COPD, no further details available), insulin-dependent diabetes mellitus type 2, diabetic chronic kidney injury (no further details available), arterial hypertension, hyperlipidaemia, liver cirrhosis, cerebral artery disease with bilateral arteria carotid interna stenosis, and peripheral artery disease stage IV (leading to the mentioned amputation). Until 2007, chronic nicotine abuse was present (50 pack years). Chronic medication included ASS 100 mg/d, doxazosin 4 mg/d, simvastatin 40 mg/d, amlodipine 5 mg/d, linagliptin 5 mg/d, and insulin. In August 2021, the patient had been hospitalised and treated for urosepsis.

### 3.4. Further Diagnostics

**Figure 1:**
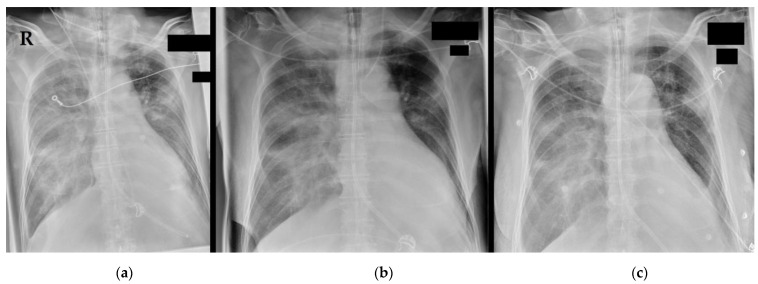
Chest X-ray at admission to the emergency department: Chest X-rays at (**a**) admission, (**b**) +14 h, and (**c**) +34 h. The endotracheal tube was detectable cranial of the carina, a gastric tube was placed, and from (**b**), a central venous catheter was inserted into the left internal jugular vein. Bilateral consolidations are compatible with pneumonic infiltrations and congestion. Bilateral pleural effusions can unfortunately not be optimally described due to the condition of the shot. The left upper lateral lobe shows the known bronchial tumour. R = right patient side.

Chest X-rays (Figure 1) showed bilateral consolidations compatible with pneumonic infiltration, with a progressive dynamic over two days’ time. A computed tomography (CT) scan (+ venous contrast medium) of the head, thorax, and abdomen revealed no further acute pathology apart from bilateral pleural effusions (up to 9 cm) and the known bronchial carcinoma (around 4 cm in diameter) of the left upper lobe. Laboratory values are presented in Table 1. In brief, creatinine values were initially within the residual range (no acute kidney injury), a troponin-T amount of 207 ng/L was regredient in the further workup, and infection markers were elevated with an initial C-reactive protein (CRP) amount of 20 mg/dL, rising to a maximum of 23 mg/dL, but eventually falling to 17 mg/dL (procalcitonin rising to a maximum of 3 ng/mL, and interleukin-6 following the dynamics of the CRP). A urine test showed no signs of urinary tract infection. Routine PCR testing delivered multiple negative results for SARS-CoV-2 and influenza but a positive test for RSV (Ct 17.8 at admission, 18.2 on day 2). Blood and urine cultures were still negative 14 days after collection.

### 3.5. Final Diagnosis and Deterioration

Due to the absence of a definitive alternative diagnosis, the CA event was deemed as hypoxic, caused by an exacerbation of the chronic pulmonary pathologies (COPD, carcinoma) either parallel to or directly through an acute RSV infection. After rewarming following TTM, sedation and relaxation were discontinued. However, the patient did not wake up, and brain stem reflexes were continuously negative, suggesting severe hypoxic brain injury. In an end-of-life discussion, the close relatives opted against further intensive care treatment, which was in accordance with the opinion of the treating multiprofessional team. Thus, multiorgan failure developed, and the patient deceased 40 h after admission to the ICU of the ED.

## 4. Discussion

We present a case of a patient with chronic pulmonary disease, complicated by an RSV infection likely triggering hypoxic CA. While it is known that endothelial dysfunction plays a major role in sepsis and also viral disease [22], we considered the systemic capillary leak syndrome resulting in CA [23], but the patient did not fulfil the criteria (missing haemoconcentration). Additionally, when applying the H score for haemophagocytic lymphohistiocytosis, which has been associated with viral disease in the past [24,25], our patient did not test positive.

Even when an exacerbation of chronic pulmonary disease including malignancy leading to CA may seem like an everyday case in clinical routine, our finding of the additional acute RSV infection proves important due to three reasons (a more detailed explanation is provided below): (1) hazard of contagion and isolation, (2) impact on post-resuscitation care, and (3) increased awareness for RSV infection in critically ill adults.

### 4.1. An Old Foe in Disguise

With RSV as a well-known pathogen in paediatric patients, it is likely to be often underestimated or overlooked elsewhere; however, multiple epidemiological and mathematical models suggest RSV as the second most frequent aetiology of viral respiratory disease in adults [14,26], with a subsequent substantial impact on global public health. Especially in people of advanced age, RSV is to be taken seriously, for instance, when assessing the data reporting over 10,000 deaths in persons over 65 years in the United States annually [3]. It is a predictable cause of epidemics of seasonal respiratory illness, and a breeding ground for complications of comorbidities or superinfections clinicians may be faced with in primary care [1,27]. In addition, with RSV being transmittable for up to 21 days and its ability to survive for several hours on various surfaces, it poses a potential hazard for healthcare workers and other patients alike. Isolation and a full set of protective wear including gowns, masks, and goggles are recommended when treating an RSV-positive patient [1,8,9,28]. With the ongoing coronavirus disease 2019 (COVID-19) pandemic, much progress in this topic has been achieved, but the question remains if protective measures are good enough or sufficiently well-executed [28]. Interestingly, it has recently been observed by the Center for Disease Control (CDC) in the United States that the typical seasonal patterns of respiratory virus outbreaks apart from COVID-19 have changed or even declined. Disrupted chains of transmission—probably due to social distancing and protective gear—have led to a prolonged absence of natural exposure to viruses such as influenza or RSV. Lower levels of population immunity may, in turn, lead to lower numbers on the one hand, while to more severe cases in those individuals who do become infected on the other [7,29]. Mattia et al. even suggested a potentially dramatic effect at times RSV epidemics should recur, with a large number of people being immunologically naive to the disease [30]. Recent mathematical models support these considerations [31,32], and other reports even consider an “immunity debt” toward various diseases induced by the current pandemic [33]. Very recent reports actually report large RSV outbreaks again, further strengthening this hypothesis [34,35,36].

### 4.2. RSV as a Possible for Cardiac Arrest

Reports on RSV as a direct causative agent for CA are scarce, but excess mortality during the winter months is well-known. As mentioned before, this has often been attributed to influenza epidemics, with the unknown dark figure of RSV potentially amounting to up to a quarter of cases [3]. Other indirect associations, namely a seasonal peak in episodes of sudden cardiac death or a higher risk of out-of-hospital cardiac arrest (OHCA) with an increasing influenza incidence have induced discussions, but associations are still too vague to draw conclusions [37,38,39]. However, viral diseases through influenza or SARS-CoV-2 lead to a prothrombotic state, potentially inducing acute coronary syndrome and pulmonary embolism, eventually leading to CA [40]. Furthermore, the direct induction of dysrhythmia has been suggested through respiratory viruses [40,41]. Naturally, the mentioned complications such as myocarditis [40], or exacerbations of comorbidities can also lead to CA—perhaps primarily caused by RSV in a number of cases, but remaining undetected and under-reported. Apart from potential cardiovascular disease induced by viral agents as an underlying factor in CA [38], the hypoxic component is probably frequent and intuitive. The treatment of asphyxia or hypoxia as a potentially reversible cause for CA is of highest priority in respective guidelines, with effective ventilation as a key therapeutic feature and low survival rates and scarce survival with neurologically favourable outcomes, highlighting the severity of the problem [42]. It is of course not directly relevant if the inducing agent of CA was RSV or any other pathogen in the acute setting of advanced life support (ALS)—at least due to missing causative treatment options. However, of utmost importance, this information could become relevant in post-resuscitation care, when—even experimental or off-label—treatments can be administered. Rising death rates in RSV cases may in fact reflect frailty or major comorbidities of affected persons; however, the reported severe cases of younger individuals, excess mortality, and healthcare costs should induce an increased awareness of the problem, leading to stronger efforts in vaccine and medication research and development [11,16,43]. Therefore, and also on the background of a population potentially increasingly becoming immunologically naive to RSV [30], we suggest routine testing for RSV, similar to routine testing for SARS-CoV-2, in cases of hypoxic CA or CA of unknown aetiology. Naturally, more cases such as the one presented by us should be collected in the future to strengthen our hypothesis. Additionally, other (co)diagnoses and comorbidities must not be overlooked when assessing similar reports.

## 5. Conclusions

A case of acute RSV infection in a patient with pre-existing chronic pulmonary disease, thus leading to CA, raises questions about the importance of RSV infection in critically ill adult patients. RSV as the causative agent for CA is possible and should be considered during evaluation after the restoration of spontaneous circulation. From a wider public health perspective, immuno-naivety for RSV caused by the COVID-19 pandemic may potentially induce a rise in cases, morbidity, and mortality in the future.

## Figures and Tables

**Table 1:** Selected laboratory values and respective dynamics throughout the stay.

BGA (Arterial)	Admission	Laboratory Values	Admission	+7 h	+11 h	+17 h	+21 h	+34 h
PH	7.3	Haemoglobin, g/dL	7.3	8.1	8.4	8.3	8.2	9.0
PCO_2_, mmHg	37	Leucocyte count, G/L	12.7	11.0	11.7	11.1	10.7	14.1
PO_2_, mmHG	130	C-reactive protein, mg/dL	19.7	21.7	22.9	22.6	21.1	16.6
K, mmol/L	3.5	Fibrinogen, mg/dL	458	486	452	476	449	483
Na, mmol/L	142	IL-6, pg/mL	252.0	76.4	41.4	21.7	22.5	13.2
Ca, mmol/L	1.3	Procalcitonin, ng/mL	0.5	1.5	2.0	2.4	2.5	2.9
Cl, mmol/L	110	LDH, U/L	410	305	243	216	209	219
Glucose, mmol/L	222	ASAT, U/L	249	196	141	100	84	74
Lactate, mmol/L	1.3	ALAT, U/L	99	103	96	92	87	84
BE, mmol/L	−9.7	Creatinine mg/dL	2.3	2.3	2.3	2.3	2.2	2.4
HCO3, mmol/L	16.5	Urea, mg/dL	41.8	43.4	46.0	46.0	45.5	50.4
Anion gap, mmol/L	19.4	GFR, mL/min/1.73 m^2^	27.7	27.9	28.2	28.5	29.4	26.9
		Hs-troponin-T, ng/L	207	197	183	154	151	149
		NT-proBNP, pg/mL	>35,000	>35,000	>35,000	>35,000	>35,000	>35,000

Selected laboratory values throughout the patient’s hospital stay. Dynamics in time are in relation to the patient’s admission to the emergency department. Decimal numbers are only given when meaningful. BGA = blood gas analysis; BE = base excess; IL = interleukin; LDH = lactate dehydrogenase; ASAT = aspartate transaminase; ALAT = alanine transaminase; GFR = glomerular filtration rate; Hs = highly sensitive; BNP = brain natriuretic peptide.

## Data Availability

Data are available from the corresponding author upon reasonable request.
